# Left Atrial Strain Correlation with Functional Capacity and Additional Prognostic Value of Speckle Tracking in Cardiac Amyloidosis: A Prospective, Single-Center Study

**DOI:** 10.3390/jcm15041337

**Published:** 2026-02-08

**Authors:** Maria Concetta Pastore, Marta Focardi, Federica Marrese, Elisa Giacomin, Gian Luca Ragazzoni, Francesca Susini, Alessandro Gozzetti, Giulia Elena Mandoli, Luna Cavigli, Elena Placuzzi, Laura Spaccaterra, Sara Rosi, Lorenzo Tanzi, Flavio D’Ascenzi, Serafina Valente, Matteo Cameli

**Affiliations:** 1 Department of Medical Biotechnologies, Division of Cardiology, University of Siena, 53100 Siena, Italy; focardim@unisi.it (M.F.);; 2 Hematology Unit, Azienda Ospedaliera Universitaria Senese, 53100 Siena, Italy

**Keywords:** cardiac amyloidosis, echocardiography, functional capacity, speckle tracking, prognosis

## Abstract

**Background**: Cardiac amyloidosis (CA) is mainly characterized by diastolic dysfunction, with gradually worsening functional capacity and poor prognosis. Left atrial (LA) strain by speckle tracking echocardiography (STE) is an index of diastolic function and heart failure (HF) symptoms. The aim of this study was to evaluate the relationship of LA strain with functional capacity in CA and the potential prognostic value of speckle tracking variables. **Methods**: In this single-center study, we prospectively enrolled consecutive outpatients with CA (n = 75). Clinical, echocardiographic evaluation, six-minute-walking-test (6MWT) and Kansas City Cardiomyopathy Questionnaire (KCCQ) were performed on the same day. The primary endpoint was the correlation between global peak atrial longitudinal strain (PALS) and NTproBNP, 6MWT score, and KCCQ. The secondary endpoint was a combination of all-cause or cardiovascular death and HF hospitalization. **Results**: Overall, 48 ATTR and 27 AL patients (74 ± 11 years, 84% male) were enrolled. Global PALS showed a significant direct correlation with N-terminal-pro-brain natriuretic peptide (NTproBNP, *p* = 0.3, *p* = 0.017) and 6MWT (*p* = 0.4, R^2^ = 0.2, *p* = 0.004), but no significant correlation with KCCQ (*p* = −0.13, *p* = 0.3). GLS showed a significant direct correlation with NTproBNP (*p* = 0.3, *p* = 0.017) but not with 6MWT and/or KCCQ. Over a mean follow up of 12 ± 3 months, 42 patients reached the combined endpoint. With ROC curves, both global PALS < 13.5% and GLS > −12% provided a good prediction of the combined endpoint (AUC = 0.72 [0.6–0.82] and 0.73 [0.63–0.83], respectively, *p* < 0.0001), higher than NTproBNP and other echocardiographic parameters. **Conclusions**: Global PALS is associated with congestion and functional capacity in CA, suggesting its role as a more objective marker of disease severity in CA. Speckle tracking parameters may be used to enhance prognostic stratification in CA.

## 1. Introduction

Cardiac amyloidosis (CA) is an infiltrative restrictive cardiomyopathy characterized by the accumulation of misfolding protein in the myocardial interstitium. Although there are more than 30 proteins that could infiltrate the myocardium, immunoglobulin-derived light chains (AL) and transthyretin (ATTR) alone account for approximately 98% of cases of CA [1].

The principal result of protein deposition is left ventricular (LV) hypertrophy, with increasing stiffness of the LV wall and first diastolic and then systolic dysfunction, clinically manifesting as heart failure (HF), with increasing worsening of functional capacity and quality of life (QoL) of patients. Demonstration of elevated left ventricular (LV) filling pressure is pivotal for the diagnosis of HF with preserved ejection fraction (HFpEF). According to the recommendations from the American Society of Echocardiography (ASE) and the European Association of Cardiovascular Imaging (EACVI), the combination of several echocardiographic parameters, including left atrial (LA) strain by speckle tracking echocardiography (STE), was proposed to evaluate LV filling pressure. When used in combination with the conventional markers, LA strain provides an additional value to predict LV filling pressure, especially in the grey zone or when one of the other standard criteria is missing [2].

STE is a validated tool in the setting of early diagnosis of CA, given that it shows a reduction of global longitudinal strain (GLS) consistent with LV damage, with a typical pattern shown by a bull’s eye representation, called apical sparing [3]. Moreover, LV diastolic dysfunction, along with direct amyloid infiltration of atrial walls, results in LA remodeling and functional impairment with significantly reduced LA reservoir strain (peak atrial longitudinal strain, PALS) and LA contraction strain (peak atrial contraction strain, PACS). However, its possible association with functional capacity and QoL in CA has not yet been investigated.

The Kansas City Cardiomyopathy Questionnaire (KCCQ) is composed of an overall summary score that mirrors functional capacity and HF symptoms burden [4] and is recommended as a measure of functional capacity in patients with HF.

The six minute walking Test (6MWT) is a functional capacity measure that has shown fair accuracy in predicting morbidity and mortality from heart or lung disease [5] and also has proven useful in big meta-analyses [6,7].

Both KCCQ and 6MWT are recommended for the evaluation of CA and are mandatory items for therapy prescription in wild-type ATTR CA.

The aim of this observational pilot study was to evaluate the correlation between LA strain and functional status (assessed as 6MWT, KCCQ and NTproBNP) in patients with CA and to assess the potential prognostic value of speckle tracking parameters in these patients.

## 2. Materials and Methods

### 2.1. Patient Population and Clinical Evaluation

In this prospective observational study, we enrolled consecutive outpatients with CA (ATTR or AL, diagnosed according to the current ESC guidelines [8]) during routine visits dedicated to CA in our tertiary level center from January to December 2023. Exclusion criteria were known coronary artery disease or prior heart failure with reduced ejection fraction, left ventricular ejection fraction <40%, severe valvular heart disease, previous cardiac surgery, non-feasible 6MWT or speckle tracking analysis, unwillingness to sign informed consent, age <18 years (central illustration).

All patients underwent a complete clinical evaluation, including 12-lead electrocardiogram, transthoracic echocardiogram (basic and advanced), serum biochemistry including N-terminal pro-brain natriuretic peptide (NT-proBNP) and high sensitivity troponin T (Hs-Tn).

On the same day of the visit, patients underwent 6MWT and 12-item version KCCQ.

For the first test, the primary measurement was the total distance walked, then we evaluated fatigue and dyspnea, measured with a modified Borg or visual analog scale. We measured arterial oxygen saturation via pulse oximetry at the beginning and at the end of the test. We evaluated the results according to the American Thoracic Statement Guidelines for the 6MWT. Healthy subjects’ 6-minutes’ walking distance range was considered to be from 400 to 700 m [9]. Patients with significant pulmonary or muscular-joint disease with potential impact on 6MWT distance or oxygen saturation evaluation were excluded.

The KCCQ printed version was administered to patients, and scores were scaled from 0 to 100 and frequently summarized in 25-point ranges, where scores represent health status as follows: 0 to 24: very poor to poor; 25 to 49: poor to fair; 50 to 74: fair to good; and 75 to 100: good to excellent [4].

Then, patients were followed for clinical endpoints with phone calls or follow up visits.

The primary endpoint was the correlation between global PALS and NTproBNP, 6MWT score and KCCQ. As the secondary endpoint, a combination of all-cause or cardiovascular death and HF hospitalization were registered during follow-up visits.

All patients gave their informed consent for study participation. The study was conducted according to the guidelines of the Declaration of Helsinki, and it was approved by the South Eastern Tuscany Ethics Committee (protocol code 19212, date of approval 28 June 2021).

### 2.2. Standard Echocardiography and Speckle Tracking Analysis

Standard echocardiography was performed by an expert operator using a fully equipped machine (Vivid E9, GE, Horten, Norway) in left lateral recumbent position with a stable ECG tracing. All parameters were measured according to the European Association of Cardiovascular Imaging (EACVI)/American Society of Ecochardiography (ASE) guidelines [10]. For each patient, the following measurements were taken: end-diastolic thickness of the ventricular septum (IVS) and of LV posterior wall (PW), end-diastolic thickness of right ventricular (RV) wall, LV end-diastolic and end-systolic volumes (EDV, ESV, respectively), and ejection fraction (EF) with biplane Simpson method. For the assessment of diastolic function, mitral peak flow velocity in early and in late diastole, during atrial contraction (E, A, respectively), pulsed TDI- derived early peak diastolic velocity at septal mitral annulus (E’) and E⁄E’ ratio, LA area and volume, and LA volume index (LAVi) were all measured. We also evaluated RV longitudinal and systolic global function by tricuspid annular plane systolic excursion (TAPSE), systolic excursion velocity (S’), and right ventricular fractional area (RVFAC).

Speckle tracking analysis was performed offline using the dedicated 2D strain software (Echopac v.206, GE, Horten, Norway) by a single experienced and independent echocardiographer, who was not directly involved in the image acquisition and blinded to other data. Dedicated views for LV, LA and RV STE, with good visualization of all chambers and a reliable delineation of the endocardial border acquired on 2D grey-scale echocardiography during three consecutive cardiac cycles with a frame rate of 40–80 frames per second and a stable ECG tracing, were used for the analysis. The endocardial border was manually traced in apical views, delineating a region of interest (ROI) of 6 segments for each view. Then, necessary manual adjustments of the ROI were performed, and the longitudinal strain curves for each segment were generated by the software.

Left ventricular global longitudinal strain (GLS) was measured using left ventricle apical four-, two-, and three-chamber views, acquired during three consecutive cardiac cycles. We manually traced the endocardial border, and then the software automatically generated the region of interest (ROI) in each apical view (four-, two-, and three chambers), which was manually corrected when needed. The left ventricle was automatically divided into 17 segments [10]. Global PALS was calculated at the end of the reservoir phase as the average of all LA segments in apical four- and two-chamber views, and using QRS as the starting point, global peak atrial contraction strain (PACS) was calculated at the beginning of LA contraction on LA strain curve as the average of all segments in apical four- and two-chamber views [11]. Right ventricular longitudinal strain (RVLS) and free-wall RVLS (fwRVLS) were assessed using the RV-focused apical four-chamber view, tracing, similar to the left ventricle, the endocardial border, adjusting, if necessary, the ROI, and obtaining, using the software, the six segments of the right ventricle, three of them for the septum and three for the free wall [11].

In patients for whom some segments were excluded for the lack of adequate tracking, strain was calculated by averaging values measured in the remaining segments.

### 2.3. Statistical Analysis

Data are expressed as means ± SD (continuous normal variables) or median [interquartile range, IQR] (continuous non-normal variables) or as counts and percentages (binary variables). The Kolmogorov–Smirnov test was used to test parameters for normality.

Patients were then divided into two groups based on ATTR or AL CA.

Differences between the groups were analyzed using independent sample Student T-tests for continuous variables (Mann–Whitney U-test for non-normally distributed variables) and Chi-squared analyses for categorical variables.

Pearsons’ coefficient was used to determine the correlation between global PALS and 6MWT score and KCCQ score in the whole population and then in the two groups.

Moreover, receiver operating characteristic (ROC) curves were applied to test the prognostic value of clinical, basic and advanced echocardiographic parameters, chosen based on previous studies and biological plausibility for the secondary endpoint. Youden index was used to identify the optimal prognostic value for the best fitted parameters.

## 3. Results

Overall, 75 patients with CA (74 ± 11 years, 48 ATTR, 27 AL, 84% male) were enrolled. Mean time from first diagnosis of CA was 6 ± 5 months. Most patients had signs or symptoms of amyloidosis systemic involvement, including in the nervous system (87%, n = 65), kidneys (48% n = 36), liver (16%, n = 12), joints (45%, n = 34), digestive tract (65%, n = 49), and bone marrow (36%, n = 27). Most patients were treated with diuretics, angiotensin converting enzyme (ACE) inhibitors or angiotensin receptor blockers (ARB) and/or mineralcorticoid receptor antagonists (MRA). Among patients with ATTR, 85% (n = 41) were treated with tafamidis for 6 ± 3 months, and the remaining 15% had contraindications due to NYHA class >2.

Mean LV ejection fraction was preserved (55 ± 9%), and LV global longitudinal strain was reduced (GLS =−12 ± 7%); 32 had an apical sparing pattern. Median global peak atrial longitudinal strain (PALS) was reduced (median [IQR] = 14 [6.5;23.5]), mean 6MWT score = 382 ± 104, and mean KCCQ score= 67 ± 24. General and clinical characteristics of the study population are shown in Table 1.

### 3.1. Standard Echocardiography

Most patients showed preserved biventricular function, with normal left ventricular (LV) ejection fraction (55 ± 9%), normal right ventricular fractional area change (RVFAC) (41 ± 10%), and normal longitudinal function of the right ventricle (TAPSE = 20 ± 8 mm).

As is typical of CA, patients had increased left ventricular wall thickness; the interventricular septum was 15 ± 3 mm and posterior wall thickness was 13 ± 3 mm. LA volume index was preserved or mildly increased (30 ± 8 mL/m^2^). Echocardiographic characteristics of the study population divided according to AL and ATTR amyloidosis are shown in Table 2.

### 3.2. STE Analysis

Most patients showed reduced LV global longitudinal strain (GLS =−12 ± 7%), 32 of them with apical sparing pattern. PALS was 14% (median [IQR] = 6.5; 23.5), and PACS was 9 ± 7%. Additionally, FWRVLS decreased (13 ± 9%). All echocardiography data in patients with ATTR and AL amyloidosis are reported in Table 2.

**Figure 1:**
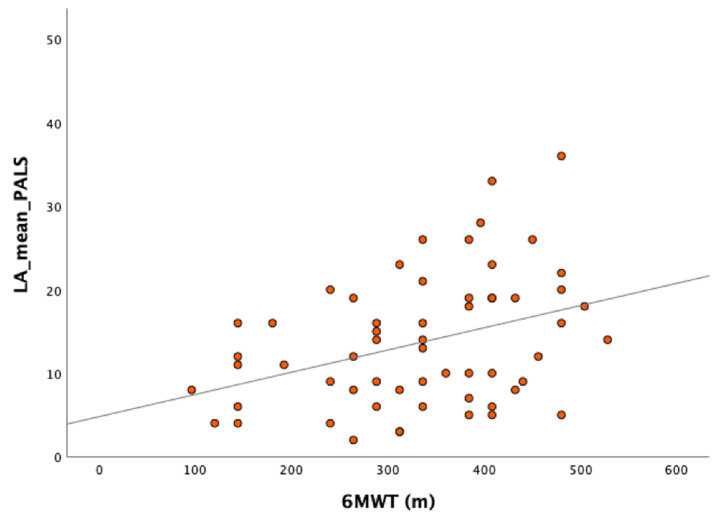
Scatter plot showing global peak atrial longitudinal strain (PALS) correlation with the six-minute walking test (6MWT).

As for the primary endpoint, global PALS showed a significant direct correlation with N-terminal pro brain natriuretic peptide (NTproBNP, *p* = 0.3, *p* = 0.017) and 6MWT (*p* = 0.4, R^2^ = 0.2, *p*-value = 0.004, Figure 1) but no significant correlation with KCCQ (*p* = −0.13, *p* = 0.3). GLS showed a significant direct correlation with N-terminal pro brain natriuretic peptide (NTproBNP, *p* = 0.3, *p* = 0.017) but not with 6MWT and/or KCCQ.

**Figure 2:**
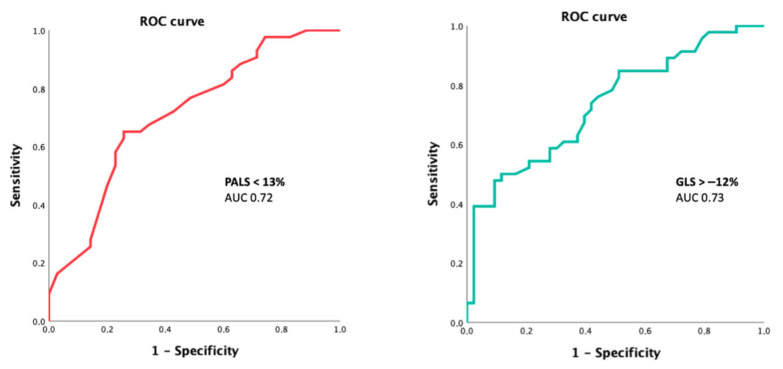
Receiver operating characteristic (ROC) curves for the prediction of the secondary endpoint by global peak atrial longitudinal strain (PALS) and left ventricular (LV) global longitudinal strain (GLS).

Over a mean follow up of 12 ± 3 months, 42 patients reached the combined endpoint (39 HF hospitalization, 3 deaths, of which 2 had cardiovascular causes). ROC curves showed that both global PALS < 13.5% and GLS > −12% provided a good prediction of the combined endpoint (AUC 0.72 [0.6–0.82] and 0.73 [0.63–0.83], respectively, *p* < 0.0001, Figure 2), higher than LAVI, TAPSE, LV EF, E/E’, free wall right ventricular strain, and NTproBNP (AUC 0.67, 0.62, 0.54, 0.65, 0.62, and 0.63, respectively).

The superior predictive value of PALS and GLS was confirmed both in AL and ATTR patients (Table 3).

## 4. Discussion

To date, our pilot study was the first to evaluate the association of global PALS and functional capacity assessed by 6MWT and KCCQ in patients with CA, showing a potential correlation with 6MWT and NTproBNP, which confirm its potential role as a marker of HF symptoms, and the absence of a correlation with KCCQ raises concerns on this parameter. Moreover, it confirmed previous findings, suggesting the potential role of speckle tracking parameters in predicting prognosis in patients with CA, although evidence in this field is still poor.

Many previous studies have focused mainly on the functional and structural consequences of amyloid infiltration within the ventricles, which appear to be the most impaired chambers due to the considerable hypertrophy. However, CA is a restrictive cardiomyopathy, mainly characterized by diastolic dysfunction. Therefore, the study of the left atrium should not be overlooked in these patients. Moreover, atrial involvement is not only due to the elevated LV filling pressure, but also to the direct infiltration of the atrial wall by misfolding proteins, causing the loss of the normal architecture of the myocardium and the increase of stiffness, as in the ventricles. 

In the previous literature, the assessment of the atria has focused mainly on atrial dimension, which is considered a marker of diastolic dysfunction [12,13]. However, deterioration in LA function occurs before structural changes; thus, the assessment of LA function could provide a more sensitive assessment of diastolic dysfunction than LAVI. In the last few years, LA strain emerged as a new, additive and earlier marker of LV diastolic dysfunction [14] and has shown its correlation with NTproBNP in HF patients [15]. Inoue et al. showed that LA reservoir and pump strain had better correlation with invasively assessed (by right heart catheterization) LV filling pressures than the standard echocardiographic parameters, with an optimal cut-off value of 18% for LA reservoir strain when defining pulmonary capillary wedge pressure of >12 mmHg as elevated [2]. Potter et al. investigated the association of LA strain with incident HF in patients with risk factors for HF with a diastolic dysfunction grading based on previously validated LA strain cut-off values, showing that incident HF significantly increased with higher diastolic disfunction grade [16].

Based on all of this evidence, the 2022 expert consensus document of the European Association of Cardiovascular Imaging for multimodality imaging assessment of HFpEF recommended the use of LA reservoir and pump strain as additional markers of LV filling pressure [17].

CA commonly presents as HFpEF, with early diastolic dysfunction in absence of relevant symptoms. In this context, the clinical implication of LA involvement in CA is of great clinical interest. A recent multicenter study, conducted on 423 consecutive patients screened for CA, showed that, among STE measures of the four chambers, PALS and PACS are the most informative to diagnose CA [18] and were particularly reduced in ATTR form. A study by our group demonstrated how LA reservoir strain may be useful in providing early diagnosis of AL CA in patients with multiple myeloma [19].

In our analysis, LA strain showed a significant difference between ATTR and AL patients, as it was lower in the first group, despite similar LA dimensions. Additionally, Versteylen et al. [20] showed that patients with ATTR CA had higher LV hypertrophy, lower LA reservoir, and pump strain independent of the other measurements of the LA dimension and function. Therefore, LA functional quantification using strain analysis may also be useful as an insight into the disease etiology and its consequences, and it could be an autonomous marker of atrial restrictive dysfunction.

In recent studies, LA strain showed its utility in recognizing CA for differential diagnosis with other cardiomyopathies; in fact, it is considerably lower in patients with CA compared to Anderson–Fabry cardiomyopathy [21]. Additionally, Monte et al., studying 100 patients, among which 33 had ATTR-CA and 34 had hypertrophic cardiomyopathy, showed that the CA group exhibited significantly impaired LA function compared to HCMs and control groups, and this impairment was consistent even in the CA subgroup with preserved ejection fraction [22].

**Figure 3:**
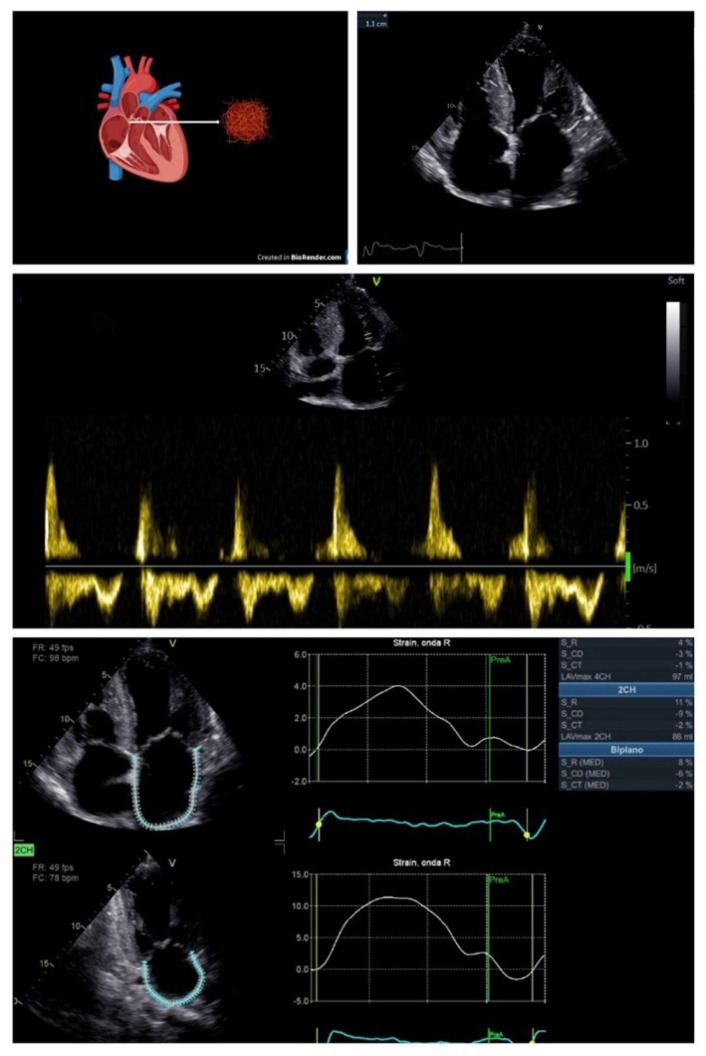
Atria infiltration by misfolding proteins causes the increase of interatrial septum thickness with restrictive doppler pattern and reduced peak atrial longitudinal strain (PALS) and peak atrial longitudinal strain (PACS).

As the primary endpoint, the present study evaluated its contribution to predict the worsening quality of life and functional capacity in patients with CA. Due to the rapid changes in clinical status characterizing patients with CA, it is important to have an objective tool to assess the worsening congestion and functional capacity in order to consider modification of the medical therapy and stricter follow up. This study demonstrated that a reduction of LA strain is associated with worse symptoms in means of lower functional capacity assessed by 6MWT and NTproBNP. In fact, as amyloid accumulates, the wall stiffness increases, with a considerable rise of LA pressure leading to high-grade diastolic dysfunction (Figure 3), with clinical impact on functional capacity and HF symptoms. On the contrary, LV GLS was not correlated with HF symptoms, probably due to the pathophysiology of CA, which mainly consists of diastolic dysfunction, with an earlier and higher burden on the left atrium typical of HFpEF [23]; therefore, the onset of symptoms in these patients is consequential to LA chronic pressure overload and incipient dysfunction.

On the other hand, our study showed the lack of correlation between LA strain and KCCQ score as a quality-of-life indicator. CA has a psychological impact on patients and their caregivers. Therefore, it is challenging to reliably assess the quality of life of these patients, as it may be influenced by their psychological status concerning the disease. In fact, even though KCCQ is the standard test recommended to assess quality of life in cardiomyopathies, many clinicians agree on the fact that, based on their experience, this tool is often not completely affordable to patients, either due to their advanced age, the unconsciousness of the importance of evaluating their personal feelings during follow-up visits, or due to high psychological burden carried by the disease, which influence the subjective perception of functional status when answering to the questionnaire. In this study, the mean KCCQ score was 67 ± 24, which includes the patients in the group ‘fair to good’. This result was not always concordant with the New York Heart Association (NYHA) class or with the daily routine that is referred to by caregivers. Therefore, some authors have already tried to find new tools for patient-reported outcomes in patients with CA [24]; however, further research is encouraged to find new tools to have a more objective assessment of QoL in CA.

The secondary endpoint of our study focused on the research on predictors of clinical endpoint in CA, for which GLS and PALS emerged as the best associated with prognosis. In previous studies, speckle tracking parameters of all cardiac chambers showed a utility to predict clinical outcome in CA [25], particularly LA strain, both in AL and ATTR [26,27,28]. Therefore, these parameters may represent easily obtainable non-invasive markers not only for CA diagnosis but also for prognostic assessment to guide therapeutic decisions.

### Limitations

Although showing promising results, our study has some limitations that should be discussed. First of all, due to the single-center nature of the study, further studies are needed to confirm these findings over larger sample sizes and a multicenter basis. Concerning the contrasting results between ATTR and AL patients, this may be due to the poor sample size of the AL group and to the diagnosis made at a very early stage of the disease in AL patients, thanks to a screening program of our center conducted in patients with multiple myeloma, which allows us to provide AL cardiac involvement at subclinical phases. Therefore, the inclusion of both ATTR and AL patients may be seen as a limitation. However, our aim was to provide real-life evidence on the utility of echocardiographic parameters to assess functional capacity and prognosis in both ATTR and AL, as the daily management in dedicated centers focuses on both types of CA.

## 5. Conclusions

The assessment of functional capacity and QoL in patients with CA is still challenging. Our preliminary results show that global PALS is associated with functional capacity and the burden of HF symptoms in ATTR and AL amyloidosis, suggesting its role as a more objective marker of disease severity in CA, but not with KCCQ. Moreover, global PALS and GLS emerged as the most accurate prognostic predictors among clinical, basic, and echocardiographic parameters in CA. Speckle tracking may provide additional useful markers of CA burden to guide clinical decision-making; however, further studies are needed to clarify the role of these parameters in the management of patients with CA and to improve the evaluation of QoL of these patients.

## Figures and Tables

**Table 1:** General and clinical characteristics of the study population.

Variables	Overall (n = 75)
Age (years)	74 ± 11
Male (%,n)	84% (36)
Weight (Kg)	72 ± 13
Height (cm)	167 ± 8
Arterial hypertension (%, n)	16 (38%)
Diabetes mellitus (%, n)	12 (5%)
Atrial fibrillation (%, n)	22 (29%)
ATTR (%, n)	64% (48)
AL (%, n)	36% (27)
Loop diuretics (%, n)	96% (72)
ACEi/ARBs (%, n)	77% (58)
Beta blockers (%, n)	48% (36)
MRA (%, n)	84% (63)

ACEi, angiotensin converting enzyme inhibitors; AL, light-chain amyloidosis ARBs, angiotensin receptor blockers; ATTR, transthyretin amyloidosis; MRA, mineralcorticoid receptor antagonists.

**Table 2:** Echocardiographic characteristics of the study population divided into two groups according to ATTR and AL amyloidosis.

Variables	Overall (n = 75)	ATTR (n = 48)	AL (n = 27)	*p*-Value
LVEF (%)	55 ± 9	55 ± 8	54 ± 6	0.103
LV IVS (mm)	15 ± 3	17 ± 3	14 ± 3	<0.0001
LV PW (mm)	13 ± 3	15 ± 2	13 ± 2	<0.0001
End-diastolic volume of LV (ml)	93 ± 18	97 ± 31	97 ± 24	0.14
E/e’_avg_	10 ± 6	13 ± 4	11 ± 7	0.73
TAPSE (mm)	20 ± 8	19 ± 4	20 ± 4	0.167
sPAP (mmHg)	30 ± 8	32 ± 9	29 ± 9	0.104
RVFAC (%)	41 ± 10	41 ± 7	46 ± 10	0.203
LAVi (mL/m^2^)	48 ± 22	51 ± 15	44 ± 16	0.092
Interatrial septum thickness (mm)	9 ± 2	9 ± 1	9 ± 2	0.142
Right ventricular free wall thickness (mm)	8 ± 2	9 ± 2	8 ± 2	0.007
GLS (%)	−14 ± 7	−12 ± 4	−16 ± 6	0.002
PALS (%)	16 ± 11	11 ± 6	20 ± 9	<0.0001
PACS (%)	9 ± 7	4 ± 5	10 ± 7	<0.0001
FWRVLS (%)	−16 ± 9	−16 ± 5	−17 ± 7	0.622

E/E’_avg_, E diastolic wave by pulsed wave Doppler/average e’ wave by tissue Doppler imaging; FWRVLS, free wall right ventricular longitudinal strain; GLS, global longitudinal strain; IVS, interventricular septum; LAVI, left atrial volume index; LV, left ventricular; LVEF, left ventricular ejection fraction; PACS, peak atrial contraction strain; PALS, peak atrial longitudinal strain; PW, posterior wall; sPAP, systolic pulmonary artery pressure; RVFAC, right ventricular fraction area change; TAPSE, tricuspid annular plane systolic excursion.

**Table 3:** Results of receiver operating characteristic (ROC) curves for the prediction of the combined endpoint in the population divided into transthyretin (ATTR) and light-chain cardiac amyloidosis (AL).

	Overall AUC	ATTR	AL
PALS	0.72 (*p* < 0.0001)	0.74 (*p* = 0.002)	0.76 (*p* < 0.001)
LV GLS	0.73 (*p* < 0.0001)	0.85 (*p* = 0.002)	0.73 (*p* = 0.007)
LAVI	0.67 (*p* = 0.06)	0.72 (*p* = 0.08)	0.62 (*p* = 0.38)
TAPSE	0.62 (*p* = 0.2)	0.73 (*p* = 0.05)	0.56 (*p* = 0.6)
LVEF	0.54 (*p* = 0.5)	0.47 (0.8)	0.61 (*p* = 0.36)
E/E’_avg_	0.65 (*p* = 0.5)	0.56 (*p* = 0.6)	0.6 (*p* = 0.4)
fwRVLS	0.62 (*p* = 0.44)	0.67 (*p* = 0.23)	0.55 (*p* = 0.6)
NTproBNP	0.63 (*p* = 0.038)	0.68 (*p* = 0.013)	0.7 (*p* = 0.05)

AUC, area under curve; E/E’_avg_, E diastolic wave by pulsed wave Doppler/average e’ wave by tissue Doppler imaging; fwRVLS, free wall right ventricular longitudinal strain; GLS, global longitudinal strain; LV, left ventricle; LVEF, left ventricular ejection fraction; PALS, peak atrial longitudinal strain; TAPSE, tricuspid annular plane systolic excursion.

## Data Availability

The raw data supporting the conclusions of this article will be made available by the authors on request.

## Abbreviations

The following abbreviations are used in this manuscript:

6MWT	6 min walking test
CA	cardiac amyloidosis
fwRVLS	free wall right ventricular longitudinal strain
HF	heart failure
HFpEF	heart failure with preserved ejection fraction
Hs-Tn	high sensitivity troponin
IVS	interventricular septum
KCCQ	Kansas city cardiomyopathy questionnaire
GLS	global longitudinal strain
LA	left atrium
LAVI	left atrial volume index
LV	left ventricle
NTproBNP	N-terminal pro brain natriuretic peptide
PACS	peak atrial contraction strain
PALS	peak atrial longitudinal strain
PW	posterior wall
QoL	quality of life
RVFAC	right ventricular fractional area change
RVLS	right ventricular longitudinal strain
STE	speckle tracking echocardiography
TAPSE	tricuspid annular plane systolic excursion
